# Identifying miRNA-mRNA regulation network of chronic pancreatitis based on the significant functional expression

**DOI:** 10.1097/MD.0000000000006668

**Published:** 2017-05-26

**Authors:** Dan Wang, Lei Xin, Jin-Huan Lin, Zhuan Liao, Jun-Tao Ji, Ting-Ting Du, Fei Jiang, Zhao-Shen Li, Liang-Hao Hu

**Affiliations:** a Department of Gastroenterology; b Digestive Endoscopy Center, Changhai Hospital, the Second Military Medical University, Shanghai, China.

**Keywords:** biomarker, chronic pancreatitis, diagnosis, miRNA, mRNA, risk gene

## Abstract

**Background:**

The aim of this study was to explore the underlying molecular mechanism and potential molecular biomarkers of chronic pancreatitis (CP) and construct a miRNA-mRNA regulation network.

**Methods:**

To explore the involvement of miRNAs in CP, we downloaded the miRNA and mRNA expression profiles of CP patients and healthy controls and identified the differentially expressed miRNAs and genes. Functional analysis was conducted and significant pathways were utilized. Finally, the miRNA-mRNA regulation network of CP was constructed.

**Results:**

A total of 44 miRNA risk gene pathway relationships were identified, and a complex regulation network was constructed with 3 genes (*ABL1*, *MYC*, and *ANAPC13*) having the highest degree in affecting the network of CP. Importantly, 4 risk genes (*NOTCH3*, *COX5A*, *THBS1*, and KARS) and 1 risk miRNA (hsa-miR-324-5p) were identified with high prediction accuracy.

**Conclusions:**

In conclusion, we analyzed miRNAs and mRNAs expression profiles in CP, 1 risk miRNA, and 4 risk genes were identified with high prediction accuracy as biomarkers of CP. Although further evaluation in clinical study is needed, our findings provide new insights into the pathogenesis of CP and may improve the diagnosis and therapy by identifying novel targets.

## Abstract 2

Supplemental Digital Content is available in the text

## 1 Introduction

Chronic pancreatitis (CP) is an irreversible disease characterized by progressive inflammation, fibrosis of pancreas, and loss of pancreatic functions.^[1]^ Patients may experience abdominal pain and exocrine or endocrine insufficiency. CP is now mainly diagnosed with clinical manifestation and image results. However, CP patients who manifest diabetes, steatorrhea, or obvious atrophic pancreas with pancreatic calculi have progressed to the late stage of the disease. In addition, differential diagnosis between CP and pancreatic cancer is difficult in some cases.^[2]^ Therefore, an accurate and sensitive diagnostic method of CP is in urgent need.

The pathogenesis process of CP is so far unclear. Recent evidence suggests that risk factors such as alcohol, smoking, genetic or hereditary factors, and autoimmune disease^[3]^ contribute to the development of CP^[4,5]^ by modulating endogenous signal pathways such as transforming growth factor (TGF)-β1 and parathyroid hormone-related protein.^[6]^ MicroRNAs (miRNAs) were reported to play an important role in the pathogenesis of pancreatic diseases and cumulative evidence indicated that some miRNAs possess the biomarker potential for diagnostic, therapeutic, prognostic exploration of CP.^[7,8]^ For example, a panel of miRNAs including miR-31 and miR-146a played important roles in the activation of pancreatic stellate cells, which may contribute to pancreatic fibrosis.^[9]^ Several other miRNAs were supposed to be potentially involved with fibrosis during CP.^[7]^ Deregulation of the miR-217-SIRT1 pathway promotes epithelial-mesenchymal transition process in pancreatic cancer.^[10]^ MiR-21, miR-34a, miR-198, and miR-217 were significantly dysregulated in pancreatic ductal adenocarcinoma compared to healthy controls and CP.^[11]^ Although considerable progress has been made, it is still critical to discover the differentially expressed miRNA and identify the target mRNAs of miRNAs to elucidate the mechanism of miRNA regulation and construct the miRNA-mRNA regulation network of CP.

In the present study, we comprehensively analyzed the miRNA and mRNA expression profiles of patients with CP, and then obtained the risk genes and miRNAs. Subsequently, a complex miRNA-mRNA-pathway regulating network was constructed. Moreover, we compared the miRNAs expression profiles of rat CP model and human CP patients. The common differentially expressed miRNAs and their targets as well as significantly enriched pathways (sig-pathways) were also analyzed.

## 2 Methods

### 2.1 MiRNA and mRNA expression profiles of CP

The expression profile of CP was downloaded from Gene Expression Atlas (http://www.ebi.ac.uk/gxa)^[12]^ at European Bioinformatics Institute of the European Molecular Biology Laboratory (EMBL-EBI, http://www.ebi.ac.uk/). The expression profile should meet the following condition: organisms (Homo sapiens); the samples were extracted from the pancreatic tissues instead of blood or cell lines. Finally, we acquired 2 mRNA expression profiles of CP including E-MEXP-1121 and E-EMBL-6, and 1 miRNA expression profile E-TABM-664. E-MEXP-1121 was about the gene expression of pancreas ductal adenocarcinoma, normal duct cells, tumor stroma, and stroma of CP as well as of several cell lines.^[13]^ According to our research, we selected 9 CP tissue samples and 9 normal controls from E-MEXP-1121. E-EMBL-6 was the transcription profiling of human normal pancreas samples, and samples from patients with CP, pancreatic cancer, and metastatic pancreatic cancer.^[14]^ Likewise, 9 CP tissue samples and 9 normal controls were screened for our study. E-TABM-664 was about the microRNA expression patterns to differentiate pancreatic adenocarcinoma from normal pancreas and CP.^[15]^ In E-TABM-664, 33 CP and 58 normal samples were screened for our study.

### 2.2 Differentially expressed RNAs

The preprocessing of microarray data included the missing value screening and probe combination for the same gene, where the multiple probe ID would be averaged as a unique value. For the multiple mRNA expression profiles integrated from several studies, the “MetaDE” package^[16]^ of R/Bioconductor was applied to identify differentially expressed genes(DEGs), and “limma” package^[17]^ of R/Bioconductor was used to identify differentially expressed miRNAs (DEmiRs) for the single study. The miRNAs or mRNAs with *P* value <.05 were regareded as the DEmiRs or DEGs.

### 2.3 Identification of miRNAs targets and the risk pathways

The validated miRNA-target interactions were obtained from 3 sources: miRecords,^[18]^ Tarbase_v6.0,^[19]^ and miRTarBase.^[20]^ Using gene set enrichment analysis (GSEA) software, the enriched KEGG pathways (http://www.genome.jp/kegg/) were identified based on the mRNA expression profile of CP. Here, default parameters were applied. A pathway was regarded as a risk pathway of CP, only if it was statically significant (enrichment *P* value < .1) in >50% of all of the datasets related to CP. Besides, we conducted the functional enrichment analysis for the targets of DEmiRs using the Database for Annotation, Visualization, and Integrated Discovery (DAVID) tool (http://david.abcc.ncifcrf.gov/).^[21]^ Functional enrichment was assessed to be over-represented for Gene Ontology term in biological process category and pathway with *P* value < .05.

### 2.4 Identification of risk genes

To explore the gene components for risk function deregulation and further provide a bridge to dissect the underlying regulation of the miRNAs, we identified risk genes. Two conditions were required to obtain strict risk genes for CP. First, the genes must be the core risk components of the sig-pathways, which could be obtained by GSEA. Core genes contribute much to the enriched pathway; therefore, the abnormal expression of core gens has great impact on the pathway and biological process. Second, the genes were differentially expressed in CP patients compared with normal controls. Finally, the intersection of core genes and DEGs were considered as the risk genes.

### 2.5 Detection of risk miRNAs for risk functions

To recognize the significant roles which miRNAs play in the dysfunctions of CP patients, risk miRNAs were identified based on the enrichment of validated miRNA-target genes. We acquired all the genes in sig-pathways that were enriched by the GSEA for the mRNA expression profile of CP.^[22]^ According to the mRNA, the risk miRNA-mediated pathways were dissected by hypergeometric distribution. Considering the DEmiRs and sig-pathways, the hypergeometric test was used according to the following formula: 
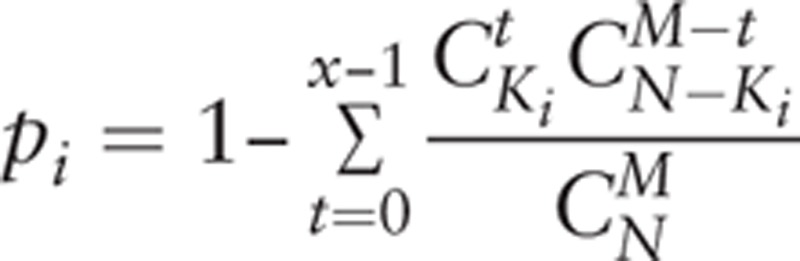
 
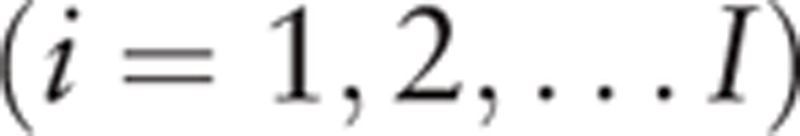


Wherein, *N* is the size of the background genes, *K*_*i*_ is the number of genes targeted by miRNA *i*, *M* is the number of genes in pathway A, *x* is the number of genes in sig-pathway A, which were also targets of DEmiR *i*, and *I* is the total number of DEmiRs. The adjusted *P* values for multiple comparisons were calculated using Benjamini approach. MiRNA-pathway pairs with *P* value <.05 were considered to be in significant relationships.

### 2.6 MiRNA-mRNA-pathway complex regulation network

We extracted the core genes and DEGs in sig-pathways, which were also targeted by DEmiRs. These genes were considered as the potential risk genes. To explore the potential functional patterns, we study the interactions among risk genes in sig-pathways.^[23]^ Human Protein Reference Database (HPRD, http://www.hprd.org/) is a database of crated proteomic information pertaining to human proteins. Currently, there were 41,327 protein-protein interactions (PPIs) in HPRD. According to our purpose, we extracted the PPIs of human. To systematically investigate the risk pathways mediated by miRNAs, we further proposed a hierarchical model to vertically explore the links among miRNAs, mRNAs, and pathways. By integrating the DEmiR-risk gene relationships, risk gene-pathway pairs, and PPI of risk genes, the miRNA-mediated pathway crosstalk network was constructed using the Cytoscape software.^[24]^

### 2.7 Microarray analysis of CP rat

We explored the miRNA expression profiles of dibutyltin dichloride (DBTC)-treated CP rat model,^[25]^ and the Affymetrix miRNA 4.0 experiment and data analysis of the 4 samples (2 CP models and 2 control models) have been completed. Total RNAs were quantified by the NanoDrop ND-2100 (Thermo Scientific) and the RNA integrity was assessed using Agilent 2100 (Agilent Technologies). The sample labeling, microarray hybridization, and washing were performed based on the manufacturer's standard protocols. Briefly, total RNA was tailed with Poly A and then labeled with Biotin. Next, the labeled RNAs were hybridized onto the microarray. After washing and staining the slides, the arrays were scanned by the Affymetrix Scanner 3000 (Affymetrix). Affymetrix GeneChip Command Console software (version4.0, Affymetrix) was used to analyze array images to get raw data and then offered RMA normalization. Next, Genespring software (version 12.5; Agilent Technologies) was used to process the following data.^[26]^ Probes that at least 100.0% of samples in any 1 condition of 2 conditions have flags in “P” were chosen for further data analysis. Differentially expressed miRNAs were then identified through fold change as well as *P* value calculated using *t* test. The threshold set for up- and downregulated genes was a fold change≥2.0 and a *P* value≤.05. The study was approved by the Ethics Committee of Shanghai Changhai Hospital. All experiments were performed in accordance with the approved guidelines and regulations, including any relevant details.

## 3 Results

### 3.1 Differentially expressed mRNAs and miRNAs in CP patients

By combining the mRNA expression profiles of E-MEXP-1121 and E-EMBL-6, 18 CP samples and 18 normal samples were acquired to study the function of mRNA acting in CP tissues. Besides, 33 CP samples and 58 normal samples were extracted to explore the roles of miRNA in the development of CP. Comparing the CP samples with the normal samples, we acquired 624 DEGs and 305 DEmiRs. Clustering analysis indicated the DEGs and DEmiRs could be differentially expressed between CP and normal samples (Supplementary Figure 1).

### 3.2 Function analysis of genes and miRNAs in CP patients

After the GSEA analysis, a total of 73 downregulated pathways and 84 upregulated pathways were identified (Supplementary Table 1) and 40 sig-pathways were acquired (Supplementary Fig. 2A). The genes in CP were significantly involved in the extracellular matrix (ECM) receptor interaction, focal adhesion, prion diseases, cell adhesion molecules cams, and viral myocarditis. Besides, the DEGs closely regulated the processes of vasculature development, cell migration, blood vessel development, and so on (Supplementary Fig. 2B).

Subsequently, targets of miRNAs were obtained from the miRecords,^[18]^ Tarbase_v6.0,^[19]^ and miRTarBase.^[20]^ There were 38,884 interactions including 771 miRNAs and 12445 target genes in total. For the DEmiRs, 2246 miRNA-targets were acquired including 78 DEmiRs and 1990 targets. After the function enrichment for the targets of DEmiRs, it came out that these DEmiRs mainly regulated Pathways in cancer, selenoamino acid metabolism, ribosome, and p53 signaling pathway (Supplementary Fig. 2C). Meanwhile, DEmiRs targeted genes mediating the regulation of protein metabolic process, RNA metabolic process, cell cycle, and so on (Supplementary Fig. 2D).

Besides, by integrating analysis of mRNA and miRNA expression profiles, we found that 142 targets were differentially expressed, which were targeted by 27 DEmiRs and consisted of 166 DEmiR-DEG pairs. In the 166 DEmiR-DEG pairs, 76 pairs downregulated and 90 pairs upregulated the target genes (Supplementary Table 2). Functions of these differentially expressed targets were also analyzed, and the results showed that DEGs targeted by DEmiRs played roles in the cellular macromolecule metabolic process, macromolecule biosynthetic process, anatomical structure formation of morphogenesis, and so on (Supplementary Fig. 3).

### 3.3 MiRNA-pathway pairs

**Figure 1:**
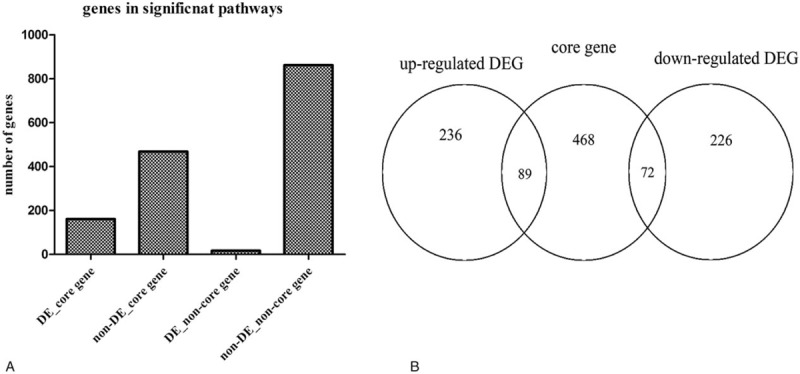
(A) The core genes in the significantly enriched pathways. (B) The number of different types of genes in sig-pathways. The relationship core genes and DEGs, DE = differentially express, DEG = differentially expressed gene.

**Figure 2:**
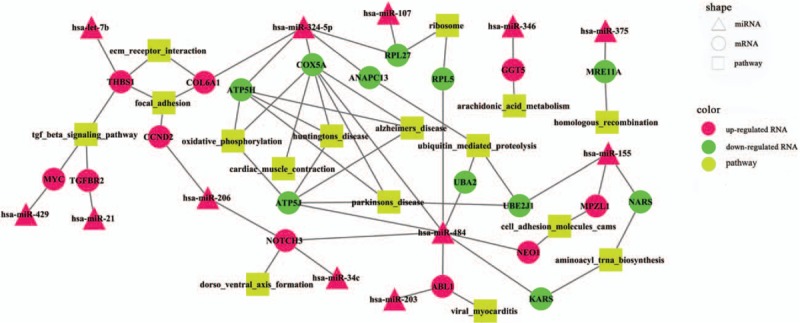
The risk miRNA-gene-pathway relationships.

In the sig-pathways, we acquired 629 core genes including 161 differentially expressed core genes and 468 nondifferentially expressed core genes, 879 noncore genes including 17 differentially expressed noncore genes, and 862 nondifferentially expressed noncore genes (Fig. 1A). Besides, 629 core genes included 89 upregulated DEGs and 72 down-regulated DEGs (Fig. 1B). Through the hypergenomic test, we acquired 33 miRNA-pathway pairs consisted of 21 DEmiRs and 17 pathways (Table 1). A total of 44 DEmiR-risk gene-pathway relationships were acquired including 12 DEmiRs, 21 risk genes, and 16 sig-pathways (Fig. 2).

**Table 1:**
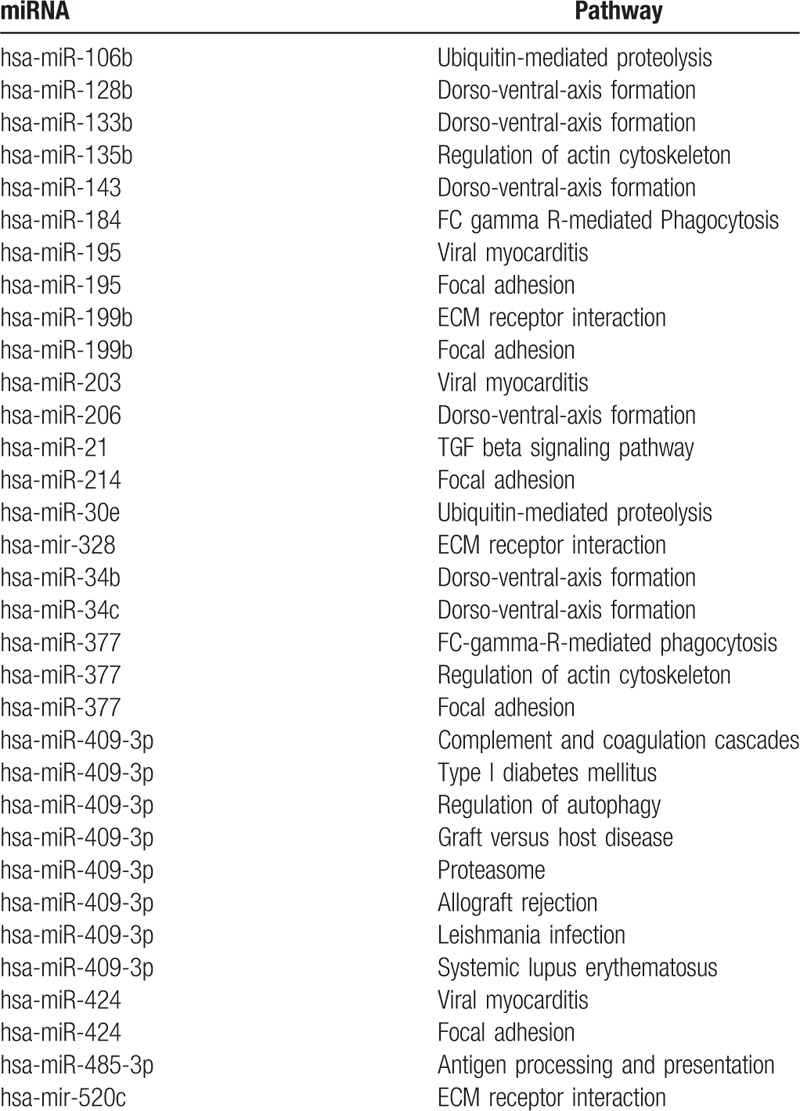
The significant miRNA-pathways pairs.

### 3.4 Analysis of miRNA-mRNA-pathway regulation network

**Figure 3:**
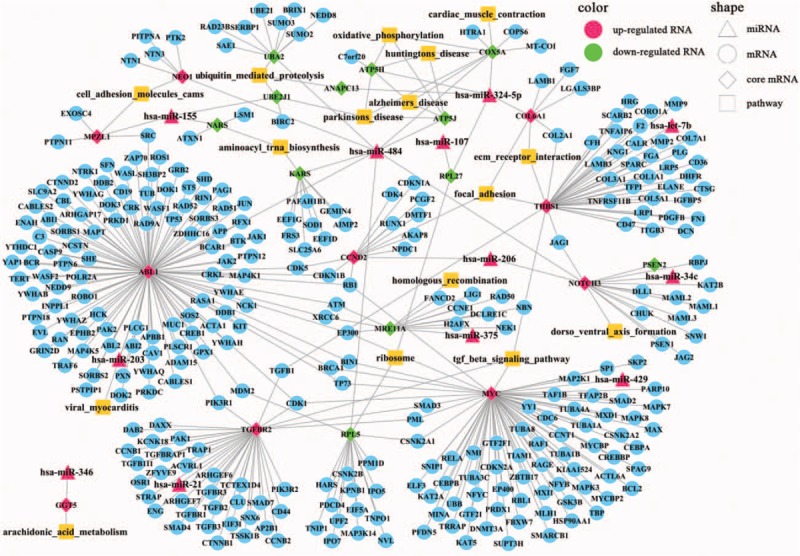
DEmiR-risk gene-pathway regulation network.

As mapping to the PPI network of HPRD database, 335 PPIs of risk genes were extracted. Combining the PPIs of risk genes and relationships among DEmiRs, risk genes, and pathways, we constructed the DEmiR-risk gene-pathway regulation network (Fig. 3).The PPI network was a scale-free network and the hub nodes would be explored according the topological characteristics. The network topological characteristics of whole network included betweenness centrality, closeness centrality, average cluster coefficient, degree, average shortest path length, and topological coefficient (Supplementary Fig. 4). In the complex network, there were 26 DEmiR-risk gene pairs, and 9 DEmiRs and risk genes in 26 DEmiR-risk gene pairs had the opposite expression trend. DEmiRs and risk genes in the rest of 17 DEmiR-risk gene pairs had the same expression tendency. For example, hsa-miR-324-5p was upregulated in the CP patients, and potentially targeted RPL27, ANAPC13, ATP5H, COX5A, and COL6A1. These genes were risk genes and differentially expressed in CP, further activated the pathways of ubiquitin-mediated proteolysis, ribosome, Parkinson disease, Huntington disease, oxidative phosphorylation, focal adhesion, and ECM receptor interaction (Fig. 3). COX5A was putatively targeted by hsa-miR-484 and hsa-miR-324-5p. Meanwhile, COX5A was downregulated in CP and was a core gene in the pathways of Parkinson disease, Huntington disease, Alzheimer disease, oxidative phosphorylation, and cardiac muscle contraction (Figs. 2 and 3). NOTCH3 was another potential biomarker and an upregulated core gene in the risk pathway of dorsoventral axis formation (Figs. 2 and 3). Besides, there were 3 upregulated DEmiRs in the CP putatively targeted NOTCH3, which were hsa-miR-206, hsa-miR-34c, and hsa-miR-484.

Moreover, we extracted topological properties of the risk genes in whole PPI network (Table 2), and found 3 genes with highest degrees (*ABL1*, *MYC* and *ANAPC13*) comparing with other risk genes (Table 2). These 3 genes affected more genes than other risk genes and may also play more important roles than other risk genes in the occurrence of CP.

**Table 2:**
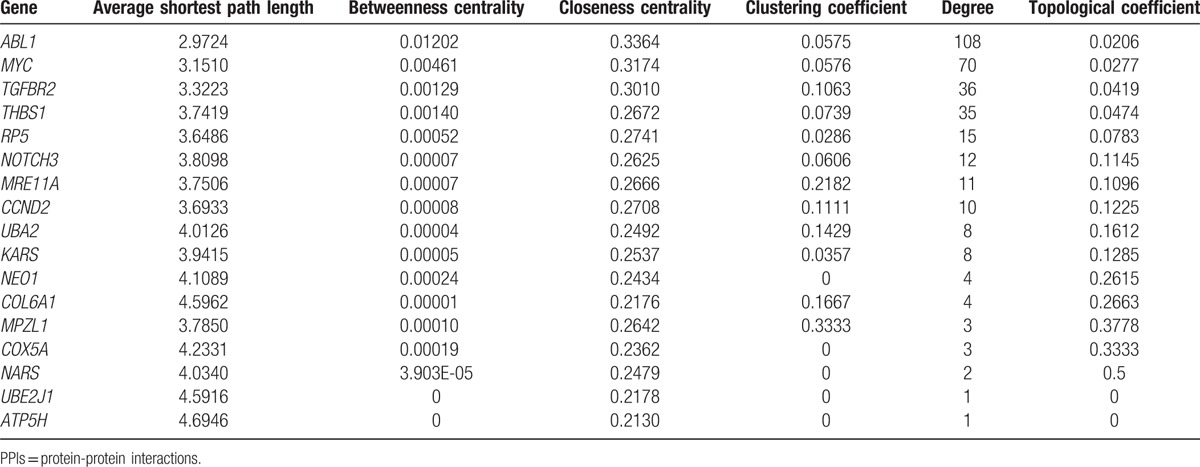
The network topological properties of risk genes in the PPI network.

**Figure 4:**
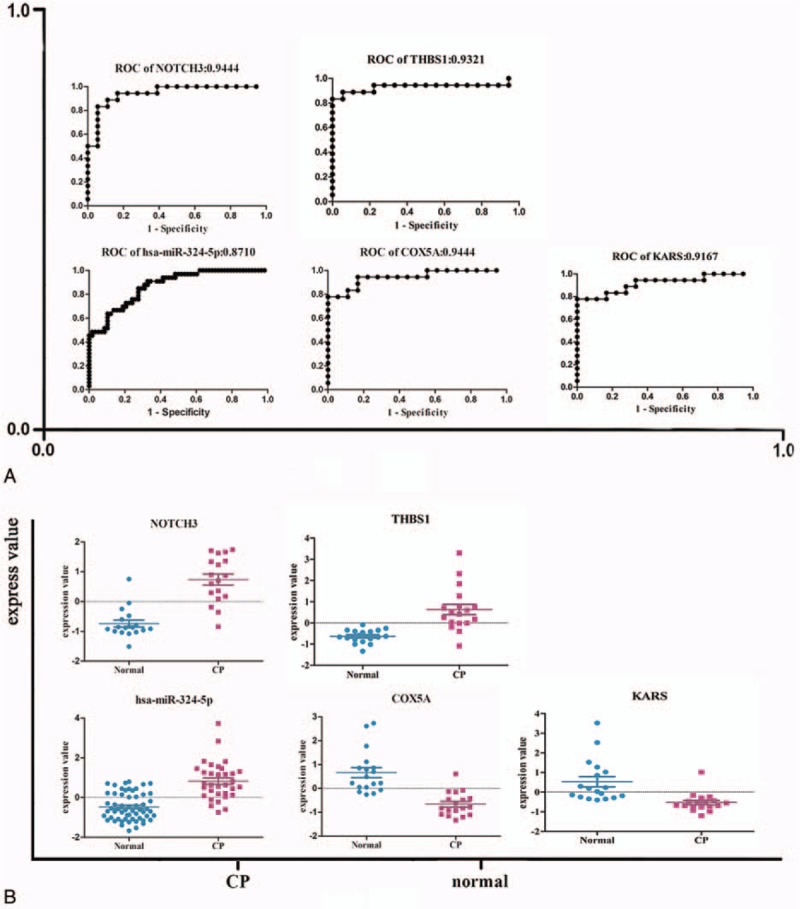
(A) Expression level and receiver-operating characteristic curve (ROC) curve for the potential biomarkers. (B) The expression level of hsa-miR-324-5p, NOTCH3,COX5A, THBS1, and KARS. The ROC curve of hsa-miR-324-5p, NOTCH3, COX5A, THBS1, and KARS.

With the 5-fold cross-validation, we assessed the prediction accuracy of DEmiRs and risk genes in the complex network (Table 3). The highest prediction accuracy miRNA was hsa-miR-324-5p, and the area under curve (AUC) was 0.871. Besides, 4 genes had the AUC >0.90, which were *NOTCH3*, *COX5A*, *THBS1*, and *KARS* (Fig. 4A). Moreover, the AUCs of *ABL1*, *MYC*, and *ANAPC13*, which ranked highest degree in the PPI network, were 0.8519, 0.8086, and 0.8981 (Tables 2 and 3). As to the expression level, the results showed that hsa-miR-324-5p, NOTCH3, and THBS1 were upregulated in the CP, whereas COX5A and KARS were downregulated in the CP (Fig. 4B). The higher prediction accuracy of hsa-miR-324-5p and COX5A showed that they may function as a motif to promote the development of CP.

**Table 3:**
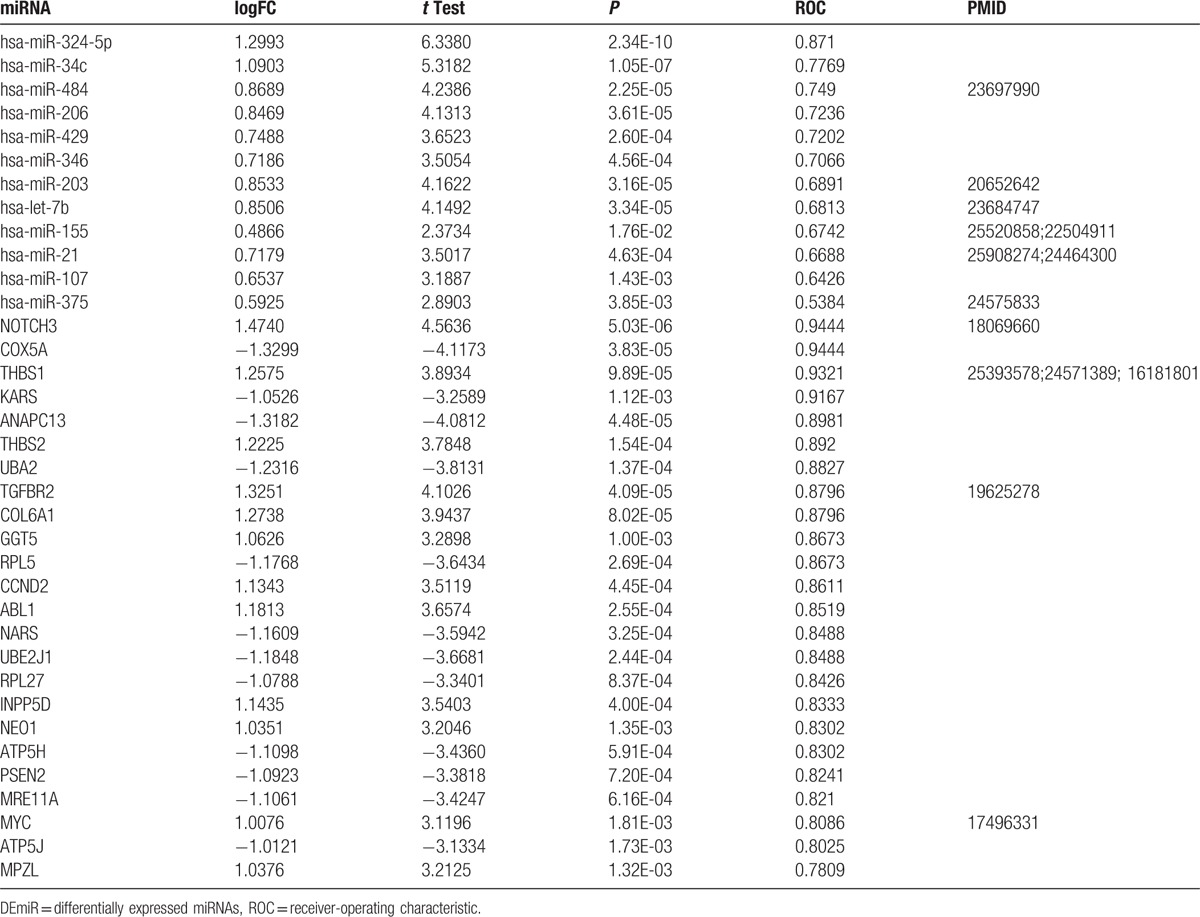
The expression level and ROC curve for the RNAs in DEmiR-risk gene-pathway relationships.

### 3.5 Comparing the differentially expressed miRNAs of rat and human

To explore whether the miRNA expression profile of rat CP model is similar with our analysis results in human CP patients, further comparison of the miRNA expression profiles of the 2 species was performed. A total of 137 DEmiRs were acquired in rat models including 4 downregulated miRNAs and 133 upregulated miRNAs (Supplementary Figure 5). There were 4 DEmiRs shared by both homo and rat including miR-182, miR-184, miR-205, and miR-431. All of the 4 miRNAs were upregulated both in homo and rat.

Target genes of DEmiRs were the intersection predicted with 2 databases (Targetscan, microRNAorg). GO analysis and KEGG analysis were applied to determine the roles of these target genes. With the DAVID, we acquired that the DEmiRs of rat took part in the regulation of apoptosis, programmed cell death, protein transport, and so on (Supplementary Fig. 6A). Besides, DEmiRs of rat got involved in the MAPK signaling pathway, pathways of cancer, adipocytokine signaling pathway, chemokine signaling pathway, and so on (Supplementary Fig. 6B). The shared DEmiRs of homo and rat mediated the negative regulation of apoptosis, negative regulation of cell death, regulation of phosphate metabolic process, and so on. The common pathways were apoptosis, colorectal cancer, neurotrophin signaling pathway, and pancreatic cancer (Supplementary Fig. 6).

## 4 Discussion

In this study, we analyzed the mRNA and miRNA expression profiles of patients with CP. The risk miRNA-gene-pathway relationships were identified including 12 risk DEmiRs, 21 risk genes, and 16 risk pathways. Twenty-one risk genes targeted by DEmiRs were acquired and were also differentially expressed in CP. Subsequently, a complex PPI regulation network was constructed. Three genes (*ABL1*, *MYC*, and *ANAPC13*) which had the highest degrees in the PPI network may play important roles in the occurrence of CP. Among the risk miRNAs, hsa-miR-324-5p had the highest prediction accuracy (0.8710) and was the potential biomarker of CP. Four of these risk genes (*NOTCH3*, *COX5A*, *THBS1*, and KARS) showing high prediction accuracy were the candidate biomarkers of CP. Moreover, we compared the miRNA expression profiles of rat and homo. Four common DEmiRs (miR-182, -184, -205, and -431) were obtained which were involved in MAPK signaling pathway etc.

In our study, 12 risk miRNAs were identified, and 7 miRNAs of them have been proved to be associated with CP or pancreatic cancer. Most of these risk miRNAs were very potentially involved in fibrosis. For example, miR-484 may regulate IL-8 expression,^[27]^ and thus is associated with fibrosis.^[28]^ MiR-34c could target notch signaling pathway^[29]^; moreover, miR-34c attenuates epithelial-mesenchymal transition and kidney fibrosis of mice^[30]^ and may target acyl-CoA synthetase long-chain family member 1 gene associated with hepatic fibrosis.^[31]^ MiR-324-5p was presented to directly target specificity protein 1 (SP1) and E26 transformation-specific 1, which play important roles in ECM signaling pathway by modulating MMP2 and MMP9 expression and activity.^[32]^ Moreover, transcription factor SP1 plays an important role in fibrosis by mediating the expression of a variety of fibrotic genes expression. Inhibition of SP1 with decoy ODN, a potent inhibitor of SP1-activated transcription, was reported to be a potential effective approach to prevent the progression of hepatic fibrosis.^[33]^ Therefore, miR-324-5p may directly target SP1 to prevent fibrosis. Furthermore, miR-324-5p is associated with NF-κB signaling pathway and thus regulates fibrosis and inflammation.^[34]^ More importantly, in our results, hsa-miR-324-5p had the highest prediction and was the potential biomarker of CP. In the complex risk miRNA-mRNA-pathway network constructed in our study, hsa-miR-324-5p putatively targeted COX5A to regulate oxidative phosphorylation,^[35]^ and may bind ANAPC13 to modulate ubiquitin-mediated-proteolysis; moreover, hsa-miR-324-5p may also got involved in ECM-receptor-interaction and focal-adhesion pathways putatively through COL6A1 gene, which is consistent with previous reports. Our findings suggested a possible molecular mechanism for CP pathogenesis.

Several proved signaling pathways were also displayed in our risk miRNA-mRNA-pathway regulating network, such as TGF-β signalingpathway, ECM signaling pathway, dorso-ventral-axis formation pathway, WNT/β-catenin signaling pathway, ubiquitin-mediated proteolysis pathway, and oxidative phosphorylation pathway. Biomolecular pathways involved in protein degradation-related pathways (lysosome, ubiquitin-mediated proteolysis, and the proteasome) were associated with the conversion of quiescent to activated pancreatic stellate cells, which is a significant event in the development of CP.^[9]^

Among the risk genes, we found that COX5A is involved in oxidative phosphorylation pathway among others; THBS1 is associated with ECM-receptor-interaction, focal adhesion, and TGF-β signaling pathway ^[36]^; KARS is correlated with aminoacyl-tRNA-biosynthesis. We also found that miR-484, -206, and -34c putatively target *NOTCH3* gene, thereby got involved in dorso-ventral-axis formation pathway. NOTCH3 is a critical factor in WNT/β-catenin signaling pathway and is also implicated in TGF-β signaling pathway associated with epithelial-mesenchymal transition.^[37]^ NOTCH3 is directly associated with fibrosis.^[38,39]^ It is markedly upregulated in fibrotic liver tissue, and may regulate the activation of hepatic stellate cells; moreover, selective inhibition of NOTCH3 prevents the hepatic fibrosis process.^[40]^ In addition, NOTCH3 is related to the occurrence of hypertension renal fibrosis.^[40,41]^ NOTCH3 expression is also reported to be elevated in the ducts of CP.^[42]^ Therefore, NOTCH3 and the other 3 risk genes may be promising risk biomarkers of CP, which need further confirmation in clinical study.

We also compared the miRNA expression profile of rats and homo. Only 4 common DEmiRs were identified both upregulated in the 2 species. On one hand, the limited number of CP model limited the accuracy of results. On the other hand, DBTC also damages other organs such as liver while inducing fibrosis in pancreas.^[43–45]^ Therefore, the pathology of DBTC-induced CP onset may be different from clinical onset of CP. As the miRNA expression profile of this rat CP model had only 4 shared DEmiRs with homo; this DBTC-treated rat CP model may not suitability for miRNA expression pattern investigation of CP.

In conclusion, we analyzed miRNAs and mRNAs expression profiles in CP, 1 risk miRNA, and 4 risk genes were identified with high prediction accuracy being as biomarkers of CP. However, further confirmation in clinical study is needed. Our findings provide new insights into the mechanisms of CP pathogenesis and may improve the diagnosis and therapy of the disease by identifying novel targets.

## Supplementary Material

Supplemental Digital Content
