# Supplementary material for: Identifying miRNA-mRNA regulation network of chronic pancreatitis based on the significant functional expression
Source: Medicine (Baltimore). 2017 May 26;96(21):e6668. doi: 10.1097/MD.0000000000006668 (PMC5457847; doi:10.1097/MD.0000000000006668)
Supplement: Supplemental Digital Content [file medi-96-e6668-s001.doc]

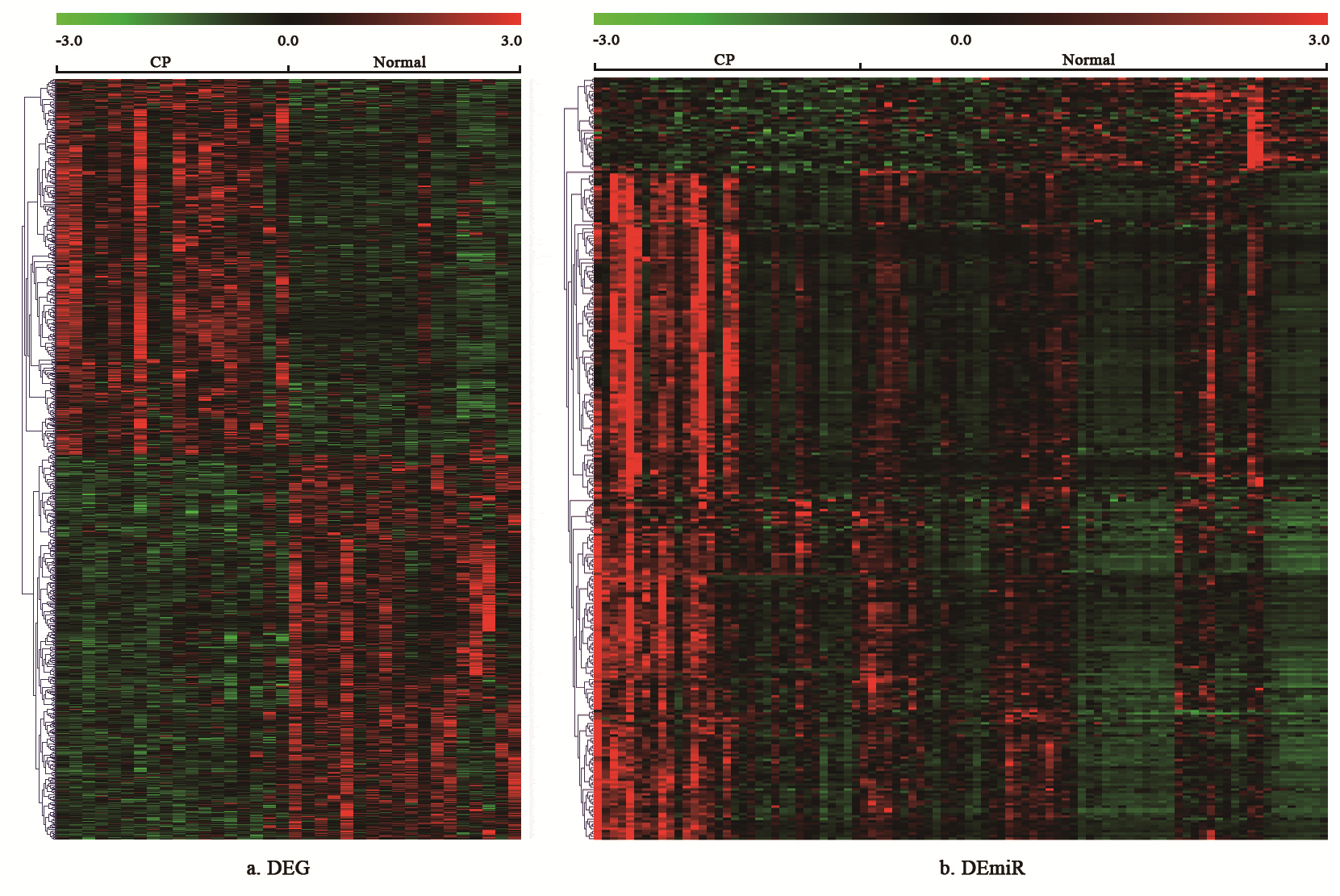


Supplementary Figure 1


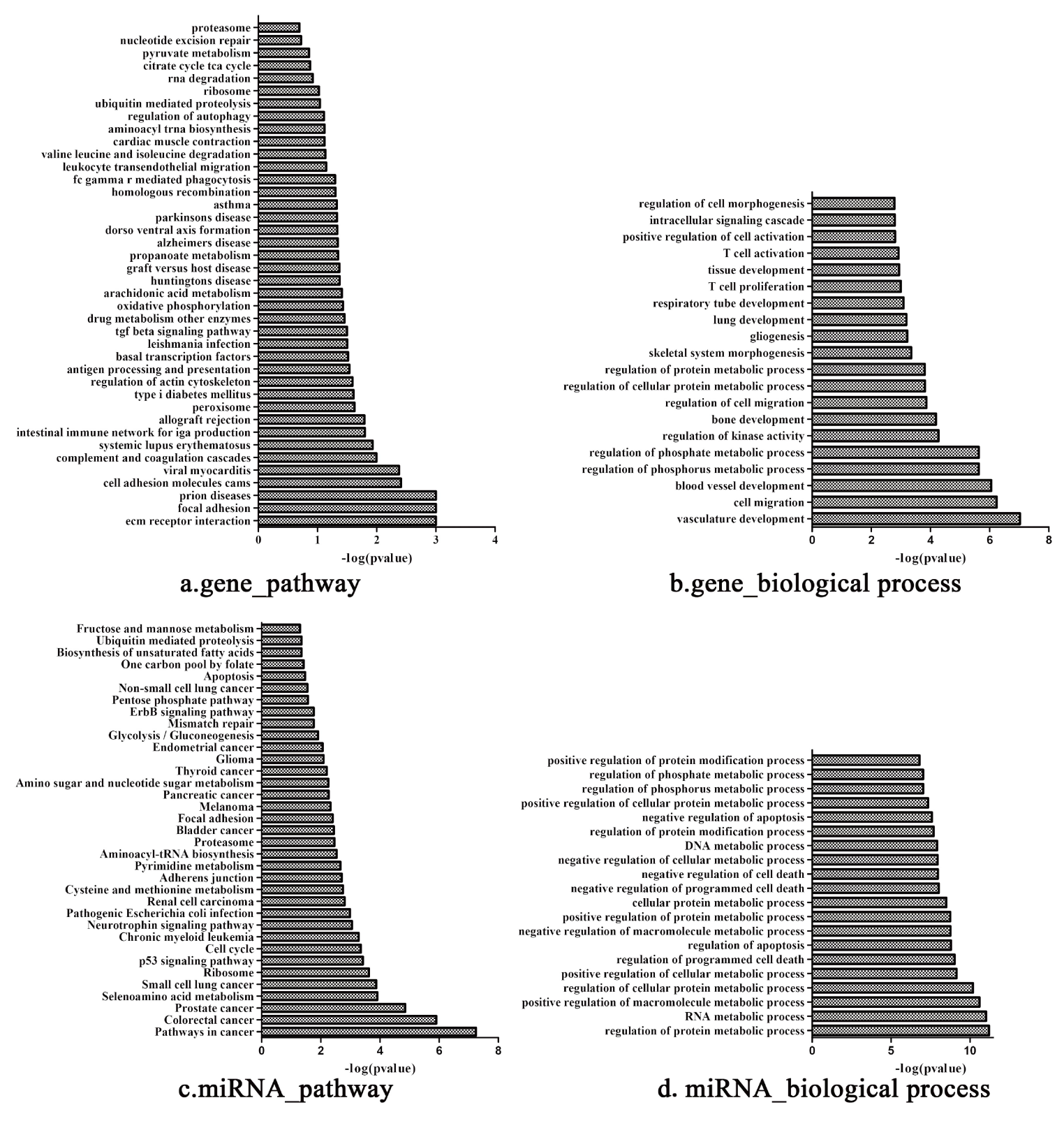


Supplementary Figure 2


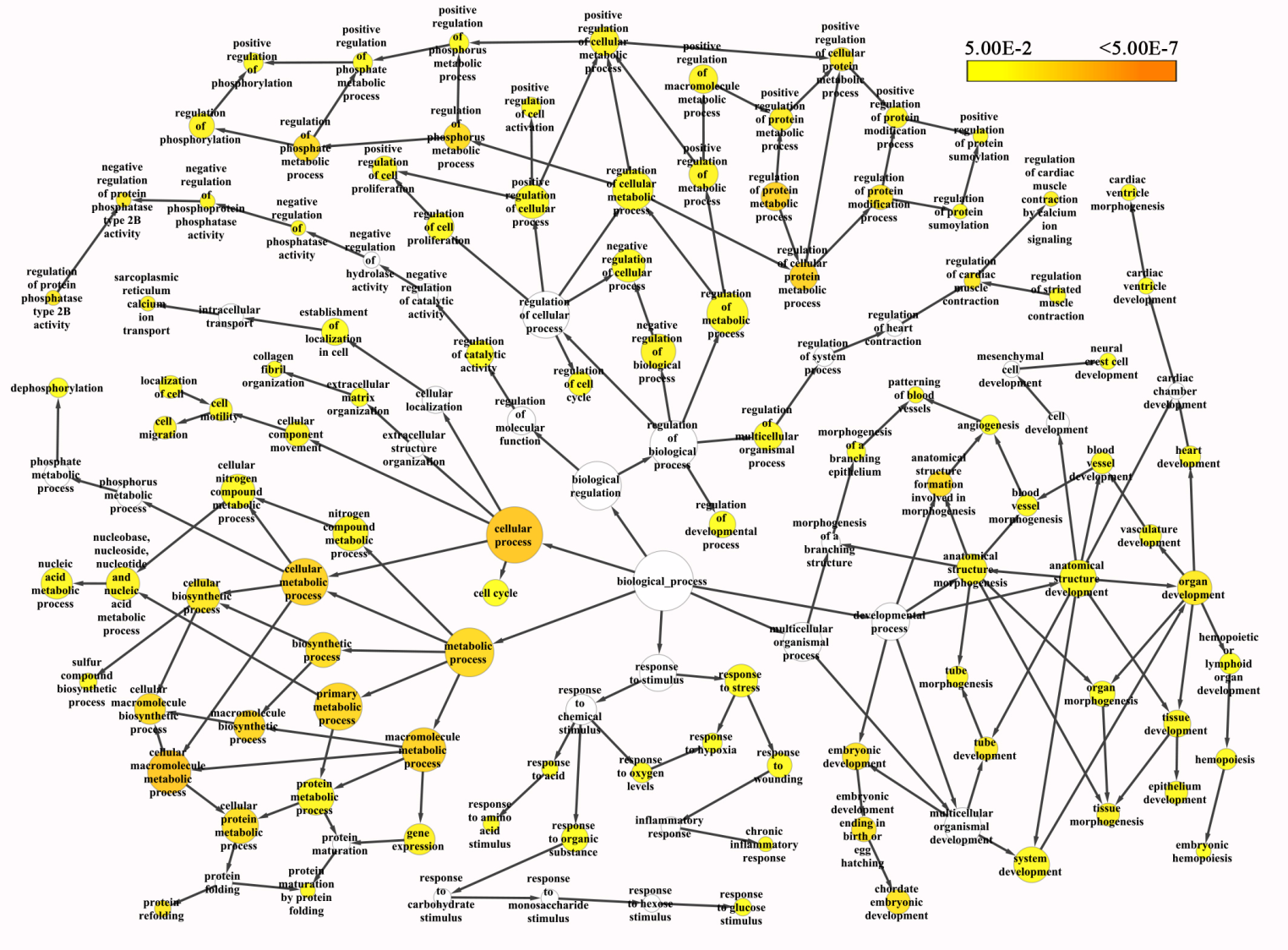


Supplementary Figure 3


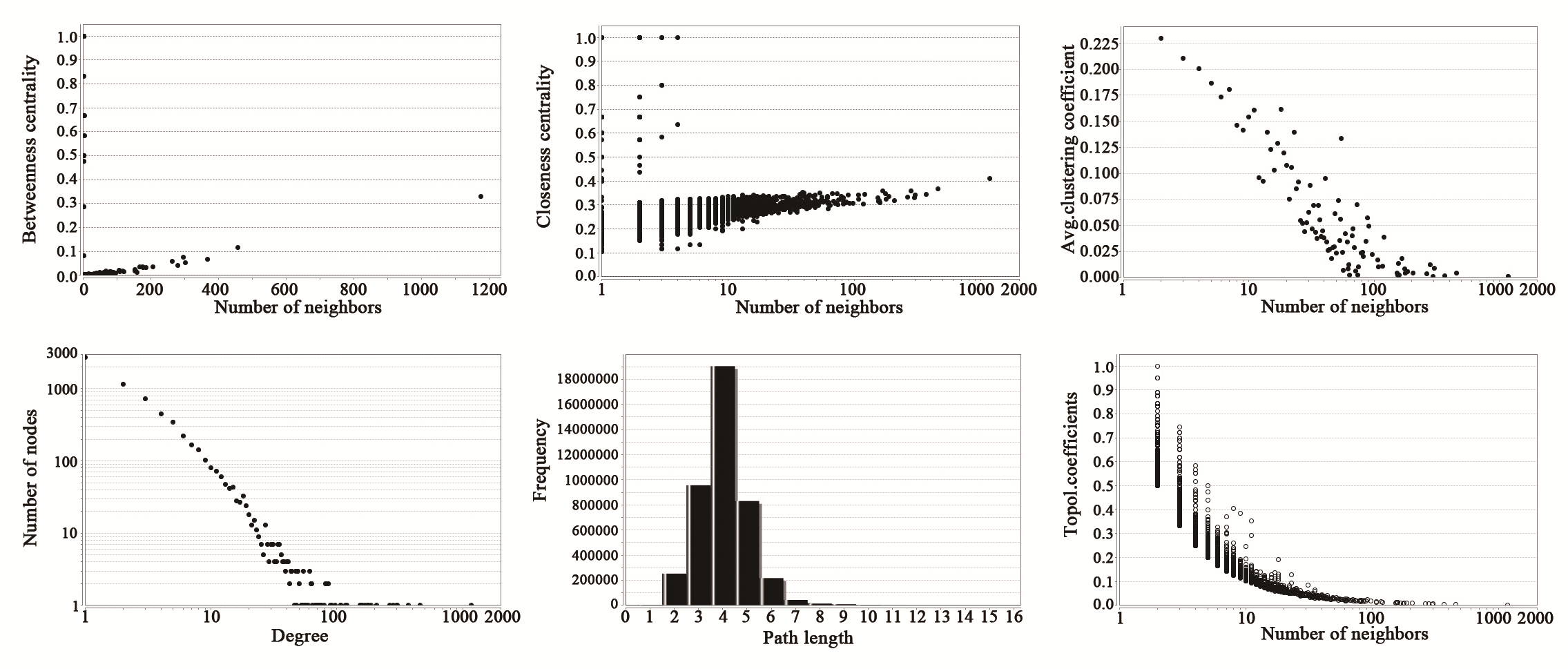


Supplementary Figure 4


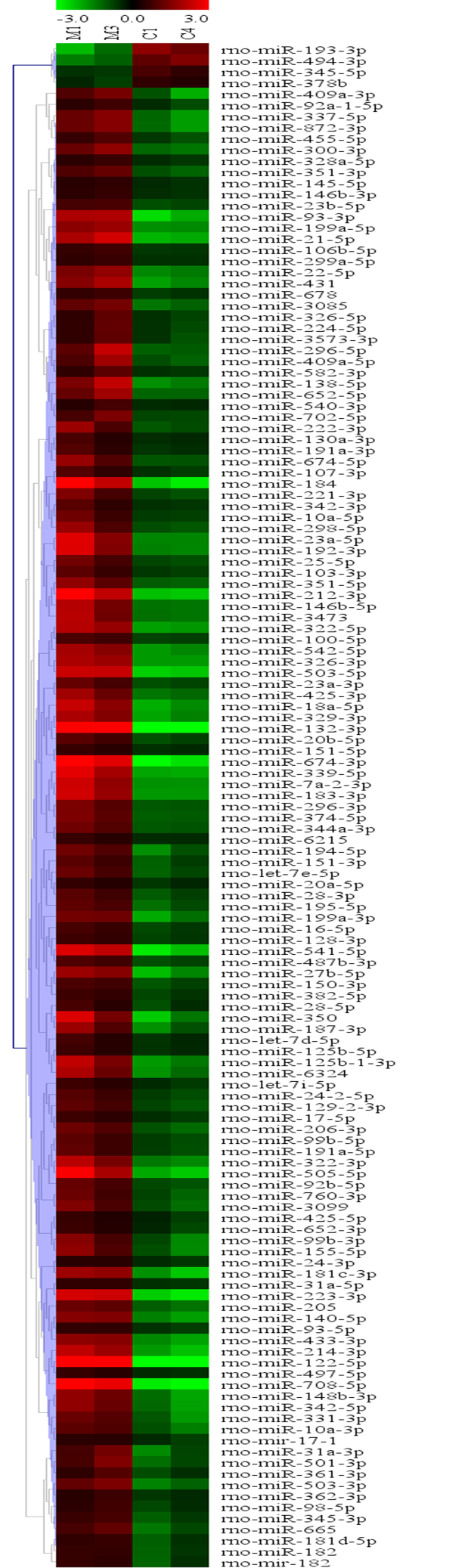


Supplementary Figure 5


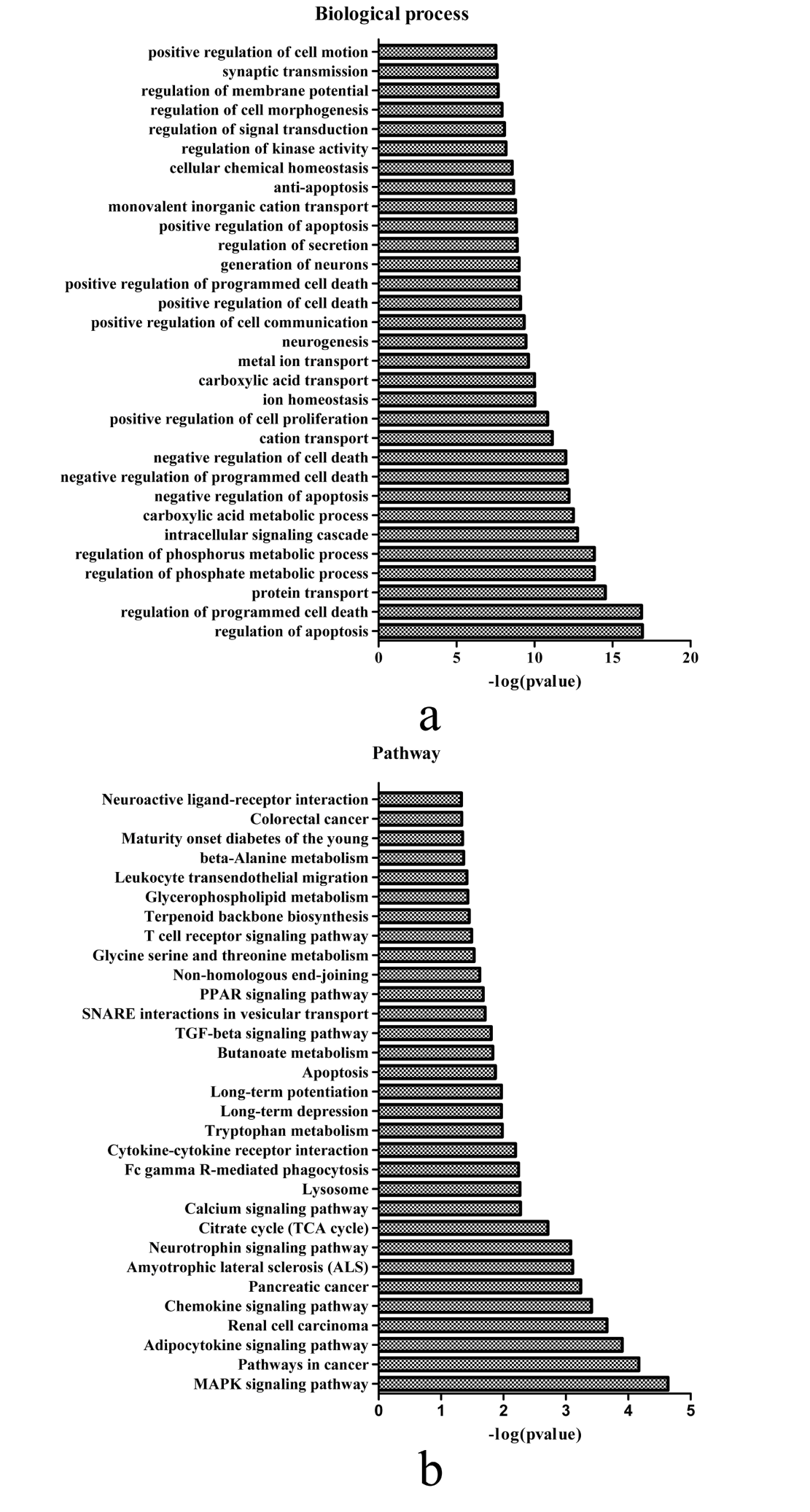


Supplementary Figure 6

Supplementary Table 1

| DEmiR-DEG Pairs | | | |
| --- | --- | --- | --- |
| miRNA | label_miRNA | gene | label_gene |
| hsa-mir-484 | up_miRNA | ABL1 | up_gene |
| hsa-mir-203 | up_miRNA | ABL1 | up_gene |
| hsa-mir-21 | up_miRNA | ACTA2 | up_gene |
| hsa-mir-484 | up_miRNA | ACTA2 | up_gene |
| hsa-mir-484 | up_miRNA | ACTB | up_gene |
| hsa-mir-326 | up_miRNA | ACTB | up_gene |
| hsa-mir-324-5p | up_miRNA | ACTB | up_gene |
| hsa-mir-484 | up_miRNA | ADAR | up_gene |
| hsa-mir-324-5p | up_miRNA | AHNAK | up_gene |
| hsa-mir-324-5p | up_miRNA | ARNT | up_gene |
| hsa-mir-107 | up_miRNA | ARNT | up_gene |
| hsa-mir-484 | up_miRNA | ATP2A2 | up_gene |
| hsa-mir-130a | up_miRNA | ATXN1 | up_gene |
| hsa-mir-21 | up_miRNA | BMPR2 | up_gene |
| hsa-mir-484 | up_miRNA | CAPZB | up_gene |
| hsa-mir-484 | up_miRNA | CBX6 | up_gene |
| hsa-mir-206 | up_miRNA | CCND2 | up_gene |
| hsa-mir-155 | up_miRNA | CEBPB | up_gene |
| hsa-mir-375 | up_miRNA | CHSY1 | up_gene |
| hsa-mir-324-5p | up_miRNA | COL14A1 | up_gene |
| hsa-mir-324-5p | up_miRNA | COL6A1 | up_gene |
| hsa-mir-375 | up_miRNA | CTDSP2 | up_gene |
| hsa-mir-375 | up_miRNA | CTGF | up_gene |
| hsa-mir-484 | up_miRNA | CYB5R3 | up_gene |
| hsa-mir-346 | up_miRNA | DGCR2 | up_gene |
| hsa-mir-484 | up_miRNA | DGCR2 | up_gene |
| hsa-mir-375 | up_miRNA | DIP2C | up_gene |
| hsa-mir-375 | up_miRNA | DPYSL3 | up_gene |
| hsa-mir-346 | up_miRNA | EFEMP2 | up_gene |
| hsa-mir-324-5p | up_miRNA | ELOVL5 | up_gene |
| hsa-mir-375 | up_miRNA | EMP1 | up_gene |
| hsa-mir-375 | up_miRNA | FKBP1A | up_gene |
| hsa-mir-324-5p | up_miRNA | FOXC1 | up_gene |
| hsa-mir-133b | up_miRNA | FSCN1 | up_gene |
| hsa-mir-107 | up_miRNA | GANAB | up_gene |
| hsa-mir-484 | up_miRNA | GANAB | up_gene |
| hsa-mir-324-5p | up_miRNA | GANAB | up_gene |
| hsa-mir-484 | up_miRNA | GCN1L1 | up_gene |
| hsa-mir-346 | up_miRNA | GGT5 | up_gene |
| hsa-mir-484 | up_miRNA | GOLGA3 | up_gene |
| hsa-mir-484 | up_miRNA | GTF3C1 | up_gene |
| hsa-mir-324-5p | up_miRNA | GTF3C1 | up_gene |
| hsa-mir-484 | up_miRNA | HCLS1 | up_gene |
| hsa-mir-140 | up_miRNA | HDAC4 | up_gene |
| hsa-mir-484 | up_miRNA | HIVEP2 | up_gene |
| hsa-mir-429 | up_miRNA | KLF11 | up_gene |
| hsa-mir-484 | up_miRNA | LAMP1 | up_gene |
| hsa-mir-375 | up_miRNA | LHFPL2 | up_gene |
| hsa-mir-223 | up_miRNA | LMO2 | up_gene |
| hsa-mir-375 | up_miRNA | MAP3K8 | up_gene |
| hsa-mir-370 | up_miRNA | MAP3K8 | up_gene |
| hsa-mir-484 | up_miRNA | MCM3AP | up_gene |
| hsa-mir-155 | up_miRNA | MPZL1 | up_gene |
| hsa-mir-429 | up_miRNA | MYC | up_gene |
| hsa-mir-484 | up_miRNA | NDST1 | up_gene |
| hsa-mir-375 | up_miRNA | NDST1 | up_gene |
| hsa-mir-484 | up_miRNA | NEO1 | up_gene |
| hsa-mir-484 | up_miRNA | NFE2L1 | up_gene |
| hsa-mir-34c | up_miRNA | NOTCH3 | up_gene |
| hsa-mir-484 | up_miRNA | NOTCH3 | up_gene |
| hsa-mir-206 | up_miRNA | NOTCH3 | up_gene |
| hsa-mir-324-5p | up_miRNA | PALM | up_gene |
| hsa-mir-107 | up_miRNA | PCSK5 | up_gene |
| hsa-mir-155 | up_miRNA | PDLIM5 | up_gene |
| hsa-mir-324-5p | up_miRNA | PPM1F | up_gene |
| hsa-mir-375 | up_miRNA | PTPN1 | up_gene |
| hsa-mir-429 | up_miRNA | RASSF2 | up_gene |
| hsa-mir-484 | up_miRNA | RFTN1 | up_gene |
| hsa-mir-183 | up_miRNA | RUNX1T1 | up_gene |
| hsa-mir-375 | up_miRNA | SAMD4A | up_gene |
| hsa-mir-375 | up_miRNA | SEMA3C | up_gene |
| hsa-mir-484 | up_miRNA | SERPINH1 | up_gene |
| hsa-mir-484 | up_miRNA | SKAP2 | up_gene |
| hsa-mir-155 | up_miRNA | SLC30A1 | up_gene |
| hsa-mir-484 | up_miRNA | SRF | up_gene |
| hsa-mir-484 | up_miRNA | TBC1D2B | up_gene |
| hsa-mir-21 | up_miRNA | TGFBR2 | up_gene |
| hsa-let-7b | up_miRNA | THBS1 | up_gene |
| hsa-mir-21 | up_miRNA | TIMP3 | up_gene |
| hsa-mir-324-5p | up_miRNA | TUBA1A | up_gene |
| hsa-mir-484 | up_miRNA | UCP2 | up_gene |
| hsa-mir-346 | up_miRNA | USP22 | up_gene |
| hsa-mir-126 | up_miRNA | VCAM1 | up_gene |
| hsa-mir-346 | up_miRNA | ZCCHC24 | up_gene |
| hsa-mir-200a | up_miRNA | ZEB2 | up_gene |
| hsa-mir-429 | up_miRNA | ZEB2 | up_gene |
| hsa-mir-192 | up_miRNA | ZEB2 | up_gene |
| hsa-mir-205 | up_miRNA | ZEB2 | up_gene |
| hsa-mir-346 | up_miRNA | ZFP36 | up_gene |
| hsa-mir-375 | up_miRNA | ZFP36L2 | up_gene |
| hsa-mir-324-5p | up_miRNA | ANAPC13 | down_gene |
| hsa-mir-224 | up_miRNA | API5 | down_gene |
| hsa-mir-484 | up_miRNA | ARFGEF2 | down_gene |
| hsa-mir-324-5p | up_miRNA | ATP5H | down_gene |
| hsa-mir-484 | up_miRNA | ATP5J | down_gene |
| hsa-mir-155 | up_miRNA | BET1 | down_gene |
| hsa-mir-375 | up_miRNA | C12orf29 | down_gene |
| hsa-mir-15a | up_miRNA | C14orf109 | down_gene |
| hsa-mir-122a | up_miRNA | CCNG1 | down_gene |
| hsa-mir-373 | up_miRNA | CD24 | down_gene |
| hsa-mir-484 | up_miRNA | CD2AP | down_gene |
| hsa-mir-484 | up_miRNA | CDC25A | down_gene |
| hsa-let-7b | up_miRNA | CDC25A | down_gene |
| hsa-mir-324-5p | up_miRNA | COX5A | down_gene |
| hsa-mir-484 | up_miRNA | COX5A | down_gene |
| hsa-mir-15a | up_miRNA | CREBL2 | down_gene |
| hsa-mir-375 | up_miRNA | CXADR | down_gene |
| hsa-mir-155 | up_miRNA | CYP51A1 | down_gene |
| hsa-mir-324-5p | up_miRNA | DAP3 | down_gene |
| hsa-mir-324-5p | up_miRNA | DEAF1 | down_gene |
| hsa-let-7b | up_miRNA | DSP | down_gene |
| hsa-mir-375 | up_miRNA | EBAG9 | down_gene |
| hsa-mir-484 | up_miRNA | EEF2 | down_gene |
| hsa-mir-484 | up_miRNA | EIF4B | down_gene |
| hsa-mir-375 | up_miRNA | FKBP1B | down_gene |
| hsa-mir-373 | up_miRNA | GBAS | down_gene |
| hsa-mir-484 | up_miRNA | GHITM | down_gene |
| hsa-mir-324-5p | up_miRNA | HNRNPH2 | down_gene |
| hsa-mir-484 | up_miRNA | HNRNPH2 | down_gene |
| hsa-mir-375 | up_miRNA | HOMER2 | down_gene |
| hsa-mir-375 | up_miRNA | HSP90AA1 | down_gene |
| hsa-mir-484 | up_miRNA | HSP90AA1 | down_gene |
| hsa-mir-346 | up_miRNA | IL18 | down_gene |
| hsa-mir-484 | up_miRNA | IMPA2 | down_gene |
| hsa-mir-107 | up_miRNA | INSIG1 | down_gene |
| hsa-mir-484 | up_miRNA | KARS | down_gene |
| hsa-mir-155 | up_miRNA | KIAA0776 | down_gene |
| hsa-mir-484 | up_miRNA | KIAA0895 | down_gene |
| hsa-mir-346 | up_miRNA | MAPK8IP1 | down_gene |
| hsa-mir-375 | up_miRNA | MRE11A | down_gene |
| hsa-mir-484 | up_miRNA | MRPL3 | down_gene |
| hsa-mir-484 | up_miRNA | MRPS18B | down_gene |
| hsa-mir-15a | up_miRNA | MSH2 | down_gene |
| hsa-mir-484 | up_miRNA | MTIF2 | down_gene |
| hsa-mir-155 | up_miRNA | NARS | down_gene |
| hsa-mir-375 | up_miRNA | NXT2 | down_gene |
| hsa-mir-155 | up_miRNA | PPP5C | down_gene |
| hsa-mir-375 | up_miRNA | PRDX3 | down_gene |
| hsa-mir-484 | up_miRNA | PROSC | down_gene |
| hsa-mir-484 | up_miRNA | PSMC2 | down_gene |
| hsa-mir-375 | up_miRNA | RBBP8 | down_gene |
| hsa-mir-484 | up_miRNA | RNF167 | down_gene |
| hsa-mir-324-5p | up_miRNA | RPL27 | down_gene |
| hsa-mir-107 | up_miRNA | RPL27 | down_gene |
| hsa-mir-484 | up_miRNA | RPL5 | down_gene |
| hsa-mir-324-5p | up_miRNA | SAP18 | down_gene |
| hsa-mir-484 | up_miRNA | SAP18 | down_gene |
| hsa-mir-484 | up_miRNA | SEPHS2 | down_gene |
| hsa-mir-375 | up_miRNA | SERP1 | down_gene |
| hsa-mir-484 | up_miRNA | SET | down_gene |
| hsa-mir-375 | up_miRNA | SET | down_gene |
| hsa-let-7b | up_miRNA | SLC1A4 | down_gene |
| hsa-mir-484 | up_miRNA | SLC1A4 | down_gene |
| hsa-mir-324-5p | up_miRNA | SLC25A3 | down_gene |
| hsa-mir-375 | up_miRNA | SLC35A3 | down_gene |
| hsa-mir-324-5p | up_miRNA | SNX7 | down_gene |
| hsa-mir-484 | up_miRNA | SURF2 | down_gene |
| hsa-mir-484 | up_miRNA | TKT | down_gene |
| hsa-mir-324-5p | up_miRNA | TRAP1 | down_gene |
| hsa-mir-484 | up_miRNA | UBA2 | down_gene |
| hsa-mir-155 | up_miRNA | UBE2J1 | down_gene |
| hsa-mir-375 | up_miRNA | UBE3A | down_gene |
| hsa-mir-375 | up_miRNA | UCHL3 | down_gene |
| hsa-mir-375 | up_miRNA | USP1 | down_gene |
| hsa-mir-375 | up_miRNA | WTAP | down_gene |
| hsa-mir-484 | up_miRNA | YWHAE | down_gene |

Supplementary Table 2

| NAME | GS<br> follow link to MSigDB | GS DETAILS | SIZE | ES | NES | NOM p-val | FDR q-val | FWER p-val | RANK AT MAX | LEADING EDGE |  |
| --- | --- | --- | --- | --- | --- | --- | --- | --- | --- | --- | --- |
| KEGG_ECM_RECEPTOR_INTERACTION | KEGG_ECM_RECEPTOR_INTERACTION | Details ... | 77 | 0.627087 | 2.015424 | 0 | 0.003215 | 0.004 | 820 | tags=36%, list=10%, signal=40% | |
| KEGG_CELL_ADHESION_MOLECULES_CAMS | KEGG_CELL_ADHESION_MOLECULES_CAMS | Details ... | 103 | 0.531686 | 1.906841 | 0.003899 | 0.021474 | 0.034 | 1632 | tags=49%, list=19%, signal=59% | |
| KEGG_FOCAL_ADHESION | KEGG_FOCAL_ADHESION | Details ... | 175 | 0.48958 | 1.878647 | 0 | 0.022419 | 0.055 | 1196 | tags=33%, list=14%, signal=37% | |
| KEGG_VIRAL_MYOCARDITIS | KEGG_VIRAL_MYOCARDITIS | Details ... | 60 | 0.557792 | 1.796644 | 0.004184 | 0.054247 | 0.15 | 1144 | tags=42%, list=14%, signal=48% | |
| KEGG_PRION_DISEASES | KEGG_PRION_DISEASES | Details ... | 31 | 0.53802 | 1.796348 | 0 | 0.043755 | 0.151 | 1263 | tags=35%, list=15%, signal=42% | |
| KEGG_COMPLEMENT_AND_COAGULATION_CASCADES | KEGG_COMPLEMENT_AND_COAGULATION_CASCADES | Details ... | 61 | 0.592433 | 1.752395 | 0.010081 | 0.062256 | 0.217 | 1304 | tags=46%, list=15%, signal=54% | |
| KEGG_ALLOGRAFT_REJECTION | KEGG_ALLOGRAFT_REJECTION | Details ... | 32 | 0.588529 | 1.738123 | 0.016097 | 0.063007 | 0.249 | 874 | tags=38%, list=10%, signal=42% | |
| KEGG_SYSTEMIC_LUPUS_ERYTHEMATOSUS | KEGG_SYSTEMIC_LUPUS_ERYTHEMATOSUS | Details ... | 62 | 0.48556 | 1.710283 | 0.011719 | 0.075306 | 0.306 | 1332 | tags=37%, list=16%, signal=44% | |
| KEGG_LEISHMANIA_INFECTION | KEGG_LEISHMANIA_INFECTION | Details ... | 59 | 0.551247 | 1.688919 | 0.031447 | 0.083251 | 0.364 | 1890 | tags=51%, list=22%, signal=65% | |
| KEGG_INTESTINAL_IMMUNE_NETWORK_FOR_IGA_PRODUCTION | KEGG_INTESTINAL_IMMUNE_NETWORK_FOR_IGA_PRODUCTION | Details ... | 38 | 0.573852 | 1.671329 | 0.015842 | 0.090689 | 0.402 | 749 | tags=29%, list=9%, signal=32% | |
| KEGG_TYPE_I_DIABETES_MELLITUS | KEGG_TYPE_I_DIABETES_MELLITUS | Details ... | 37 | 0.527091 | 1.663353 | 0.02449 | 0.090654 | 0.433 | 874 | tags=35%, list=10%, signal=39% | |
| KEGG_GRAFT_VERSUS_HOST_DISEASE | KEGG_GRAFT_VERSUS_HOST_DISEASE | Details ... | 30 | 0.599308 | 1.620077 | 0.042596 | 0.122769 | 0.546 | 874 | tags=43%, list=10%, signal=48% | |
| KEGG_ANTIGEN_PROCESSING_AND_PRESENTATION | KEGG_ANTIGEN_PROCESSING_AND_PRESENTATION | Details ... | 67 | 0.43616 | 1.590722 | 0.028986 | 0.14691 | 0.609 | 1029 | tags=30%, list=12%, signal=34% | |
| KEGG_ASTHMA | KEGG_ASTHMA | Details ... | 26 | 0.58936 | 1.575123 | 0.047228 | 0.153921 | 0.657 | 1462 | tags=50%, list=17%, signal=60% | |
| KEGG_REGULATION_OF_ACTIN_CYTOSKELETON | KEGG_REGULATION_OF_ACTIN_CYTOSKELETON | Details ... | 159 | 0.364023 | 1.539611 | 0.025532 | 0.184674 | 0.73 | 1313 | tags=28%, list=16%, signal=32% | |
| KEGG_DORSO_VENTRAL_AXIS_FORMATION | KEGG_DORSO_VENTRAL_AXIS_FORMATION | Details ... | 15 | 0.575549 | 1.539421 | 0.046185 | 0.173251 | 0.731 | 853 | tags=27%, list=10%, signal=30% | |
| KEGG_FC_GAMMA_R_MEDIATED_PHAGOCYTOSIS | KEGG_FC_GAMMA_R_MEDIATED_PHAGOCYTOSIS | Details ... | 76 | 0.43544 | 1.525444 | 0.05042 | 0.180925 | 0.757 | 2173 | tags=49%, list=26%, signal=65% | |
| KEGG_ARACHIDONIC_ACID_METABOLISM | KEGG_ARACHIDONIC_ACID_METABOLISM | Details ... | 37 | 0.458214 | 1.516334 | 0.03876 | 0.183147 | 0.777 | 1259 | tags=35%, list=15%, signal=41% | |
| KEGG_TGF_BETA_SIGNALING_PATHWAY | KEGG_TGF_BETA_SIGNALING_PATHWAY | Details ... | 75 | 0.401478 | 1.510312 | 0.031712 | 0.179603 | 0.779 | 1385 | tags=29%, list=16%, signal=35% | |
| KEGG_LEUKOCYTE_TRANSENDOTHELIAL_MIGRATION | KEGG_LEUKOCYTE_TRANSENDOTHELIAL_MIGRATION | Details ... | 89 | 0.422663 | 1.494756 | 0.071429 | 0.188847 | 0.799 | 1429 | tags=34%, list=17%, signal=40% | |
| KEGG_AUTOIMMUNE_THYROID_DISEASE | KEGG_AUTOIMMUNE_THYROID_DISEASE | | 42 | 0.454168 | 1.46064 | 0.075697 | 0.224551 | 0.845 | 874 | tags=29%, list=10%, signal=32% | |
| KEGG_DILATED_CARDIOMYOPATHY | KEGG_DILATED_CARDIOMYOPATHY | | 76 | 0.37589 | 1.420975 | 0.04277 | 0.274913 | 0.889 | 1429 | tags=28%, list=17%, signal=33% | |
| KEGG_LYSOSOME | KEGG_LYSOSOME | | 100 | 0.362037 | 1.396884 | 0.092784 | 0.300621 | 0.913 | 1652 | tags=36%, list=20%, signal=44% | |
| KEGG_ARRHYTHMOGENIC_RIGHT_VENTRICULAR_CARDIOMYOPATHY_ARVC | KEGG_ARRHYTHMOGENIC_RIGHT_VENTRICULAR_CARDIOMYOPATHY_ARVC | | 63 | 0.373287 | 1.386215 | 0.092369 | 0.30611 | 0.918 | 1429 | tags=29%, list=17%, signal=34% | |
| KEGG_B_CELL_RECEPTOR_SIGNALING_PATHWAY | KEGG_B_CELL_RECEPTOR_SIGNALING_PATHWAY | | 66 | 0.377701 | 1.379283 | 0.058577 | 0.305014 | 0.923 | 2020 | tags=41%, list=24%, signal=53% | |
| KEGG_AXON_GUIDANCE | KEGG_AXON_GUIDANCE | | 101 | 0.324497 | 1.369963 | 0.064909 | 0.309691 | 0.93 | 1497 | tags=27%, list=18%, signal=32% | |
| KEGG_HEMATOPOIETIC_CELL_LINEAGE | KEGG_HEMATOPOIETIC_CELL_LINEAGE | | 81 | 0.382653 | 1.353059 | 0.119919 | 0.324866 | 0.936 | 1400 | tags=30%, list=17%, signal=35% | |
| KEGG_HYPERTROPHIC_CARDIOMYOPATHY_HCM | KEGG_HYPERTROPHIC_CARDIOMYOPATHY_HCM | | 71 | 0.350269 | 1.335625 | 0.075 | 0.344412 | 0.946 | 1429 | tags=25%, list=17%, signal=30% | |
| KEGG_ACUTE_MYELOID_LEUKEMIA | KEGG_ACUTE_MYELOID_LEUKEMIA | | 53 | 0.365509 | 1.313935 | 0.122449 | 0.371246 | 0.956 | 2143 | tags=43%, list=25%, signal=58% | |
| KEGG_VASCULAR_SMOOTH_MUSCLE_CONTRACTION | KEGG_VASCULAR_SMOOTH_MUSCLE_CONTRACTION | | 85 | 0.35069 | 1.313411 | 0.132911 | 0.360142 | 0.957 | 2038 | tags=41%, list=24%, signal=54% | |
| KEGG_NOTCH_SIGNALING_PATHWAY | KEGG_NOTCH_SIGNALING_PATHWAY | | 31 | 0.440608 | 1.30909 | 0.153846 | 0.3558 | 0.959 | 1218 | tags=32%, list=14%, signal=38% | |
| KEGG_CHEMOKINE_SIGNALING_PATHWAY | KEGG_CHEMOKINE_SIGNALING_PATHWAY | | 147 | 0.336539 | 1.301745 | 0.163223 | 0.356333 | 0.96 | 2306 | tags=44%, list=27%, signal=60% | |
| KEGG_JAK_STAT_SIGNALING_PATHWAY | KEGG_JAK_STAT_SIGNALING_PATHWAY | | 117 | 0.335205 | 1.288834 | 0.145161 | 0.368201 | 0.967 | 1585 | tags=29%, list=19%, signal=35% | |
| KEGG_TIGHT_JUNCTION | KEGG_TIGHT_JUNCTION | | 95 | 0.284717 | 1.250575 | 0.119097 | 0.423964 | 0.981 | 1015 | tags=20%, list=12%, signal=22% | |
| KEGG_SMALL_CELL_LUNG_CANCER | KEGG_SMALL_CELL_LUNG_CANCER | | 81 | 0.314404 | 1.245844 | 0.13786 | 0.420696 | 0.982 | 1683 | tags=28%, list=20%, signal=35% | |
| KEGG_PRIMARY_IMMUNODEFICIENCY | KEGG_PRIMARY_IMMUNODEFICIENCY | | 32 | 0.357915 | 1.180417 | 0.216162 | 0.539946 | 0.997 | 1165 | tags=25%, list=14%, signal=29% | |
| KEGG_SPHINGOLIPID_METABOLISM | KEGG_SPHINGOLIPID_METABOLISM | | 23 | 0.363073 | 1.176358 | 0.260417 | 0.534625 | 0.997 | 1395 | tags=26%, list=16%, signal=31% | |
| KEGG_PATHWAYS_IN_CANCER | KEGG_PATHWAYS_IN_CANCER | | 276 | 0.248001 | 1.176 | 0.18107 | 0.521011 | 0.997 | 1433 | tags=21%, list=17%, signal=24% | |
| KEGG_MAPK_SIGNALING_PATHWAY | KEGG_MAPK_SIGNALING_PATHWAY | | 213 | 0.25656 | 1.175954 | 0.183267 | 0.507674 | 0.997 | 1988 | tags=32%, list=23%, signal=41% | |
| KEGG_GAP_JUNCTION | KEGG_GAP_JUNCTION | | 71 | 0.29487 | 1.171135 | 0.228 | 0.503422 | 0.997 | 1988 | tags=32%, list=23%, signal=42% | |
| KEGG_MELANOGENESIS | KEGG_MELANOGENESIS | | 72 | 0.292999 | 1.169697 | 0.219561 | 0.493653 | 0.997 | 1257 | tags=24%, list=15%, signal=27% | |
| KEGG_TOLL_LIKE_RECEPTOR_SIGNALING_PATHWAY | KEGG_TOLL_LIKE_RECEPTOR_SIGNALING_PATHWAY | | 83 | 0.295665 | 1.161344 | 0.242424 | 0.499001 | 0.997 | 2031 | tags=37%, list=24%, signal=49% | |
| KEGG_N_GLYCAN_BIOSYNTHESIS | KEGG_N_GLYCAN_BIOSYNTHESIS | | 28 | 0.351804 | 1.140858 | 0.284314 | 0.526894 | 0.998 | 688 | tags=21%, list=8%, signal=23% | |
| KEGG_NATURAL_KILLER_CELL_MEDIATED_CYTOTOXICITY | KEGG_NATURAL_KILLER_CELL_MEDIATED_CYTOTOXICITY | | 99 | 0.29257 | 1.140587 | 0.271047 | 0.515621 | 0.998 | 1144 | tags=22%, list=14%, signal=25% | |
| KEGG_ETHER_LIPID_METABOLISM | KEGG_ETHER_LIPID_METABOLISM | | 20 | 0.340711 | 1.134636 | 0.270563 | 0.515657 | 0.999 | 1635 | tags=45%, list=19%, signal=56% | |
| KEGG_ADHERENS_JUNCTION | KEGG_ADHERENS_JUNCTION | | 66 | 0.298828 | 1.129536 | 0.286598 | 0.513626 | 0.999 | 1377 | tags=27%, list=16%, signal=32% | |
| KEGG_FC_EPSILON_RI_SIGNALING_PATHWAY | KEGG_FC_EPSILON_RI_SIGNALING_PATHWAY | | 64 | 0.306071 | 1.116939 | 0.334677 | 0.525356 | 0.999 | 2020 | tags=41%, list=24%, signal=53% | |
| KEGG_CHRONIC_MYELOID_LEUKEMIA | KEGG_CHRONIC_MYELOID_LEUKEMIA | | 67 | 0.313588 | 1.111752 | 0.317814 | 0.52442 | 0.999 | 2020 | tags=34%, list=24%, signal=45% | |
| KEGG_PATHOGENIC_ESCHERICHIA_COLI_INFECTION | KEGG_PATHOGENIC_ESCHERICHIA_COLI_INFECTION | | 41 | 0.348191 | 1.109828 | 0.325431 | 0.518097 | 0.999 | 507 | tags=17%, list=6%, signal=18% | |
| KEGG_PHENYLALANINE_METABOLISM | KEGG_PHENYLALANINE_METABOLISM | | 16 | 0.381737 | 1.091104 | 0.342256 | 0.542638 | 0.999 | 359 | tags=19%, list=4%, signal=20% | |
| KEGG_COLORECTAL_CANCER | KEGG_COLORECTAL_CANCER | | 57 | 0.290838 | 1.038596 | 0.390086 | 0.631663 | 0.999 | 1377 | tags=21%, list=16%, signal=25% | |
| KEGG_BLADDER_CANCER | KEGG_BLADDER_CANCER | | 41 | 0.304527 | 1.035653 | 0.414583 | 0.625767 | 0.999 | 788 | tags=15%, list=9%, signal=16% | |
| KEGG_WNT_SIGNALING_PATHWAY | KEGG_WNT_SIGNALING_PATHWAY | | 107 | 0.240075 | 1.031189 | 0.407328 | 0.622186 | 0.999 | 994 | tags=15%, list=12%, signal=17% | |
| KEGG_TYROSINE_METABOLISM | KEGG_TYROSINE_METABOLISM | | 33 | 0.291385 | 1.02755 | 0.405567 | 0.61773 | 0.999 | 739 | tags=15%, list=9%, signal=17% | |
| KEGG_T_CELL_RECEPTOR_SIGNALING_PATHWAY | KEGG_T_CELL_RECEPTOR_SIGNALING_PATHWAY | | 96 | 0.254239 | 1.021068 | 0.42161 | 0.618381 | 0.999 | 2029 | tags=31%, list=24%, signal=41% | |
| KEGG_CYTOKINE_CYTOKINE_RECEPTOR_INTERACTION | KEGG_CYTOKINE_CYTOKINE_RECEPTOR_INTERACTION | | 198 | 0.269226 | 1.019265 | 0.422846 | 0.610902 | 0.999 | 1411 | tags=21%, list=17%, signal=25% | |
| KEGG_ENDOCYTOSIS | KEGG_ENDOCYTOSIS | | 128 | 0.215185 | 1.010756 | 0.4375 | 0.61665 | 0.999 | 976 | tags=15%, list=12%, signal=17% | |
| KEGG_VEGF_SIGNALING_PATHWAY | KEGG_VEGF_SIGNALING_PATHWAY | | 59 | 0.255291 | 0.989648 | 0.463074 | 0.645429 | 0.999 | 2409 | tags=42%, list=28%, signal=59% | |
| KEGG_PROSTATE_CANCER | KEGG_PROSTATE_CANCER | | 78 | 0.238878 | 0.975666 | 0.486653 | 0.661596 | 0.999 | 2020 | tags=31%, list=24%, signal=40% | |
| KEGG_BASAL_CELL_CARCINOMA | KEGG_BASAL_CELL_CARCINOMA | | 34 | 0.280056 | 0.971386 | 0.498092 | 0.658868 | 0.999 | 793 | tags=18%, list=9%, signal=19% | |
| KEGG_HISTIDINE_METABOLISM | KEGG_HISTIDINE_METABOLISM | | 19 | 0.336859 | 0.962997 | 0.533465 | 0.664251 | 0.999 | 1138 | tags=32%, list=13%, signal=36% | |
| KEGG_GLYCEROPHOSPHOLIPID_METABOLISM | KEGG_GLYCEROPHOSPHOLIPID_METABOLISM | | 47 | 0.258649 | 0.953874 | 0.544681 | 0.670613 | 0.999 | 2050 | tags=38%, list=24%, signal=50% | |
| KEGG_PHOSPHATIDYLINOSITOL_SIGNALING_SYSTEM | KEGG_PHOSPHATIDYLINOSITOL_SIGNALING_SYSTEM | | 61 | 0.258396 | 0.953553 | 0.52495 | 0.660606 | 0.999 | 2020 | tags=33%, list=24%, signal=43% | |
| KEGG_ABC_TRANSPORTERS | KEGG_ABC_TRANSPORTERS | | 34 | 0.2712 | 0.924066 | 0.584314 | 0.70785 | 1 | 1705 | tags=32%, list=20%, signal=40% | |
| KEGG_MTOR_SIGNALING_PATHWAY | KEGG_MTOR_SIGNALING_PATHWAY | | 39 | 0.262153 | 0.910099 | 0.595618 | 0.723034 | 1 | 1657 | tags=31%, list=20%, signal=38% | |
| KEGG_VASOPRESSIN_REGULATED_WATER_REABSORPTION | KEGG_VASOPRESSIN_REGULATED_WATER_REABSORPTION | | 37 | 0.247374 | 0.910031 | 0.644309 | 0.712211 | 1 | 1197 | tags=22%, list=14%, signal=25% | |
| KEGG_NOD_LIKE_RECEPTOR_SIGNALING_PATHWAY | KEGG_NOD_LIKE_RECEPTOR_SIGNALING_PATHWAY | | 47 | 0.279989 | 0.885818 | 0.588727 | 0.745469 | 1 | 1903 | tags=36%, list=22%, signal=46% | |
| KEGG_LONG_TERM_DEPRESSION | KEGG_LONG_TERM_DEPRESSION | | 57 | 0.221913 | 0.884589 | 0.656189 | 0.736816 | 1 | 1015 | tags=16%, list=12%, signal=18% | |
| KEGG_CALCIUM_SIGNALING_PATHWAY | KEGG_CALCIUM_SIGNALING_PATHWAY | | 144 | 0.210584 | 0.879282 | 0.659406 | 0.735887 | 1 | 1423 | tags=21%, list=17%, signal=25% | |
| KEGG_THYROID_CANCER | KEGG_THYROID_CANCER | | 26 | 0.252469 | 0.851471 | 0.670125 | 0.774384 | 1 | 827 | tags=15%, list=10%, signal=17% | |
| KEGG_MELANOMA | KEGG_MELANOMA | | 57 | 0.216671 | 0.85085 | 0.706349 | 0.764427 | 1 | 2020 | tags=30%, list=24%, signal=39% | |
| KEGG_ALDOSTERONE_REGULATED_SODIUM_REABSORPTION | KEGG_ALDOSTERONE_REGULATED_SODIUM_REABSORPTION | | 37 | 0.257565 | 0.845957 | 0.671233 | 0.762854 | 1 | 2589 | tags=51%, list=31%, signal=74% | |
| KEGG_GALACTOSE_METABOLISM | KEGG_GALACTOSE_METABOLISM | | 21 | 0.289875 | 0.845013 | 0.632444 | 0.754222 | 1 | 1425 | tags=29%, list=17%, signal=34% | |
| KEGG_RENIN_ANGIOTENSIN_SYSTEM | KEGG_RENIN_ANGIOTENSIN_SYSTEM | | 15 | 0.278999 | 0.828426 | 0.688492 | 0.773199 | 1 | 1527 | tags=33%, list=18%, signal=41% | |
| KEGG_RENAL_CELL_CARCINOMA | KEGG_RENAL_CELL_CARCINOMA | | 61 | 0.221125 | 0.827735 | 0.725108 | 0.764218 | 1 | 2353 | tags=38%, list=28%, signal=52% | |
| KEGG_ERBB_SIGNALING_PATHWAY | KEGG_ERBB_SIGNALING_PATHWAY | | 75 | 0.208789 | 0.813029 | 0.757202 | 0.779734 | 1 | 2342 | tags=36%, list=28%, signal=49% | |
| KEGG_PANCREATIC_CANCER | KEGG_PANCREATIC_CANCER | | 67 | 0.21485 | 0.802205 | 0.780538 | 0.786494 | 1 | 2020 | tags=33%, list=24%, signal=43% | |
| KEGG_INOSITOL_PHOSPHATE_METABOLISM | KEGG_INOSITOL_PHOSPHATE_METABOLISM | | 44 | 0.20864 | 0.791299 | 0.797189 | 0.793186 | 1 | 1103 | tags=18%, list=13%, signal=21% | |
| KEGG_ADIPOCYTOKINE_SIGNALING_PATHWAY | KEGG_ADIPOCYTOKINE_SIGNALING_PATHWAY | | 57 | 0.197508 | 0.779876 | 0.856 | 0.801054 | 1 | 1805 | tags=32%, list=21%, signal=40% | |
| KEGG_GLIOMA | KEGG_GLIOMA | | 53 | 0.199709 | 0.753327 | 0.89375 | 0.830045 | 1 | 857 | tags=11%, list=10%, signal=13% | |
| KEGG_ENDOMETRIAL_CANCER | KEGG_ENDOMETRIAL_CANCER | | 47 | 0.186663 | 0.718719 | 0.913232 | 0.865068 | 1 | 2363 | tags=34%, list=28%, signal=47% | |
| KEGG_NEUROTROPHIN_SIGNALING_PATHWAY | KEGG_NEUROTROPHIN_SIGNALING_PATHWAY | | 106 | 0.164997 | 0.699115 | 0.962656 | 0.877018 | 1 | 2276 | tags=35%, list=27%, signal=47% | |
| KEGG_TYPE_II_DIABETES_MELLITUS | KEGG_TYPE_II_DIABETES_MELLITUS | | 40 | 0.186236 | 0.678893 | 0.93254 | 0.889194 | 1 | 2037 | tags=33%, list=24%, signal=43% | |
| KEGG_LONG_TERM_POTENTIATION | KEGG_LONG_TERM_POTENTIATION | | 58 | 0.128071 | 0.503498 | 0.998028 | 0.98378 | 1 | 1988 | tags=26%, list=23%, signal=34% | |
